# Primed Pluripotent Cell Lines Derived from Various Embryonic Origins and Somatic Cells in Pig

**DOI:** 10.1371/journal.pone.0052481

**Published:** 2013-01-11

**Authors:** Jin-Kyu Park, Hye-Sun Kim, Kyung-Jun Uh, Kwang-Hwan Choi, Hyeong-Min Kim, Taeheon Lee, Byung-Chul Yang, Hyun-Jong Kim, Hak-Hyun Ka, Heebal Kim, Chang-Kyu Lee

**Affiliations:** 1 Department of Agricultural Biotechnology, Animal Biotechnology Major, and Research Institute for Agriculture and Life Science, Seoul National University, Seoul, Republic of Korea; 2 National Institute of Animal Science, RDA, Suwon, Republic of Korea; 3 Department of Biological Resources and Technology, Yonsei University, Wonju, Republic of Korea; University of Cincinnati, United States of America

## Abstract

Since pluripotent embryonic stem cell (ESC) lines were first derived from the mouse, tremendous efforts have been made to establish ESC lines in several domestic species including the pig; however, authentic porcine ESCs have not yet been established. It has proven difficult to maintain an ESC-like state in pluripotent porcine cell lines due to the frequent occurrence of spontaneous differentiation into an epiblast stem cell (EpiSC)-like state during culture. We have been able to derive EpiSC-like porcine ESC (pESC) lines from blastocyst stage porcine embryos of various origins, including *in vitro* fertilized (IVF), *in vivo* derived, IVF aggregated, and parthenogenetic embryos. In addition, we have generated induced pluripotent stem cells (piPSCs) via plasmid transfection of reprogramming factors (*Oct4*, *Sox2*, *Klf4,* and *c-Myc*) into porcine fibroblast cells. In this study, we analyzed characteristics such as marker expression, pluripotency and the X chromosome inactivation status in female of our EpiSC-like pESC lines along with our piPSC line. Our results show that these cell lines demonstrate the expression of genes associated with the Activin/Nodal and FGF2 pathways along with the expression of pluripotent markers Oct4, Sox2, Nanog, SSEA4, TRA 1–60 and TRA 1–81. Furthermore all of these cell lines showed in vitro differentiation potential, the X chromosome inactivation in female and a normal karyotype. Here we suggest that the porcine species undergoes reprogramming into a primed state during the establishment of pluripotent stem cell lines.

## Introduction

Pluripotent stem cells are capable of differentiating into all three embryonic germ layers and maintain their pluripotency and self-renewal capacity over a long period of time [1]. Since pluripotent cells were first derived from the inner cell mass (ICM) of mouse blastocysts [2], attempts to establish embryonic stem (ES) cell lines have been tried from various mammal species, including pigs [3], cattle [4], rats [5], primates [6] and humans [1].

Among these various ES cell lines, different characteristics concerning morphology, signaling pathways and cell surface marker antigens are observed across species [7]–[9]. A recent study has reported that these pluripotent stem cells exist in one of two forms and can be categorized according to their pluripotent state. The first is a “naïve” state, which is characterized by small, round or dome-shaped colony morphologies, LIF and BMP4 signaling pathways, the expression of stage-specific embryonic antigen-1 (SSEA-1) as a cell surface marker and two active X chromosomes in female; mouse ES cells (mESCs) represent this type of ES cell. A second “primed” state has also been described and is possible in mouse epiblast stem cells (mEpiSCs) or human ES cells (hESCs) [10]–[12]. These primed state pluripotent stem cells display flattened monolayer colony morphologies, FGF and Activin/Nodal signaling pathways, expressions of SSEA4, TRA 1–60 and TRA 1–81 surface marker antigens and X chromosome inactivation in female [10], [12], [13]_ENREF_13.

It has been suggested that the porcine model provides the most ideal non-primate system for clinical research regarding potential human therapeutic use, an observation based on immunological and physiological similarities with humans. In addition, porcine embryonic stem cells (pESCs) would provide powerful experimental tools, such as the production of transgenic pigs and xenografting [14]. However, for many domestic species including the pig, authentic ESCs have not yet been categorized. Given the prospective advantages and current limitations, many reviewers have recently emphasized important details that must be considered for the establishment of validated pESCs. It has been noted that defining the optimum stage of embryonic development for stem cell derivation is key to obtaining stable ES cell lines, together with elucidation of pluripotent markers and the key signaling pathways that regulate the pluripotency of pESCs. Furthermore, an understanding of species-specific mechanisms and optimal culture conditions must be achieved in order to maintain stable ES cell lines [9], [15]–[17]. Considering these challenges, in recent years, several research groups have reported putative pESCs from embryos produced *in vivo*, *in vitro* fertilization, parthenogenetic activation and somatic cell nuclear transfer [18]–[24]. It has been identified that these pESC lines showed EpiSC-like characteristics such as flattened monolayer colony morphologies and activin/nodal signaling pathway [25]. We have also attempted to establish pluripotent cell lines from porcine embryos; however, like many others, we are so far unable to derive what could be described as authentic pESC lines. However, during our research, we have been able to derive EpiSC-like pESC lines from various porcine blastocysts derived from *in vivo*, IVF, IVF aggregation, and parthenogenetic activation.

Recently published papers have reported that pluripotent cells can be induced from somatic cells using four commonly cited reprogramming genes (*Oct4, Sox2, Klf4,* and c-*Myc*)[26]–[32]_ENREF_17. Especially, West and colleagues reported porcine induced pluripotent stem cells (piPSCs) capable of generating chimeric offspring [27], [32]. However, the piPSCs has not been characterized in detail for the pluripotent state, although their ability to produce chimeric offspring had been reported [32]. We have also been successful in deriving piPSCs using these factors and previously described methods [33].

The main purpose of the present study was to investigate characteristics such as marker expression, signaling pathways, pluripotency and self-renewal in these EpiSC-like pESC and piPSC lines. In this study, we confirmed activation of the Activin/Nodal/FGF pathway and the expressions of the pluripotency markers Oct4, Sox2, Nanog, SSEA4, TRA 1–60 and TRA 1–81 in all of our cell lines. Furthermore all of these cell lines showed in vitro differentiation potential; the X chromosome inactivation in female and a normal karyotype. Our results suggest that pluripotent stem cells derived from embryos and iPSCs derived from embryonic fibroblasts in the porcine model possess a primed pluripotent state similar to that of mEpiSCs or hESCs, rather than to that of mESCs.

## Materials and Methods

### Animal Welfare

The care and experimental use of pigs and mice was approved by the Institute of Laboratory Animal Resources, Seoul National University (SNU-200909-20).

### Collection, Production and Culture of Porcine Blastocysts

Methods for the generation of porcine blastocysts, including IVF procedures, *in vivo* collection, embryo aggregation (3X) and parthenogenesis, were performed according to previously described protocols [34]–[36]. Porcine blastocysts were cultured on mitotically inactivated mouse embryonic fibroblasts (MEFs) in pESC medium, a 50∶50 mixture of Dulbecco’s modified Eagle’s medium (DMEM low glucose, Gibco Invitrogen, USA, www.invitrogen.com) and Ham’s F10 medium (Gibco), supplemented with 15% fetal bovine serum (FBS; collected and processed in Canada; Hyclone, Logan, UT, www.hyclone.com), 2 mM glutamax (Gibco), 0.1 mM ß-mercaptoethanol (Gibco), 1x MEM nonessential amino acids (Gibco), 1x antibiotic/antimycotic (Gibco) containing cytokines, 40 ng/ml human recombinant SCF (hrSCF; R&D Systems, USA, www.rndsystems.com), and 20 ng/ml human recombinant bFGF (hrbFGF; R&D Systems). Two seeding methods were used to establish pluripotent cell lines: intact blastocyst stage embryos were either cultured directly on MEFs or were subjected to mechanical dissection under the microscope using pulled glass pipettes to separate the inner cell mass (ICM) from the trophectoderm (TE) prior to seeding. Following 5–7 days of culture, we observed EpiSC-like primary colonies derived from day 7 *in vivo*-produced and day 8 *in vitro*-hatched blastocysts. These EpiSC-like pESC colonies were mechanically dissociated into several clumps using pulled glass pipettes 10–15 days after seeding. Dissociated clumps were then re-seeded on fresh inactivated MEFs, and subsequent EpiSC-like pESC lines were routinely passaged via the pulled glass pipette method every 5–7 days. All cells were cultured in humidified conditions maintained with 5% CO_2_ at 37°C.

### Generation and Culture of piPSCs

The derivation of piPSCs was conducted using previously described methods [33]. Briefly, a pCX-OKS-2A plasmid containing *Oct4*, *Klf4*, and *Sox2* and a pCX-cMyc plasmid containing *c-Myc* were obtained from Addgene (plasmids 19771 and 19772, respectively; www.addgene.org). Plasmid DNAs were purified from transformed E-coli using a plasmid DNA purification kit (iNtRON Biotechnology, Korea, www.intronbio.com) and were introduced into porcine embryonic fibroblasts (PEFs) in a 35 mm dish with Opti-MEM (Invitrogen) in a total volume of 500 µl, consisting of 2 µg pCX-OKS-2A, 1 µg pCX-cMyc, 6 µl Lipofectamine™ LTX (Invitrogen), and 2 µl Plus™ Reagent (Invitrogen).

Plasmid transfection was performed a total of four times at two-day intervals. PEFs (2×10^5^ cells) were cultured in pESC medium on mitotically inactivated MEFs in 35 mm dishes for 2–3 weeks. Transfected PEFs were transferred daily to fresh pESC medium until colonies sufficiently large to passage were observed. EpiSC-like colonies were mechanically dissociated into several clumps using pulled glass pipettes. The resulting piPSCs were routinely passaged every 5–7 days.

### Alkaline Phosphatase (AP) Activity and Immunocytochemistry (ICC) Analysis

For AP staining of EpiSC-like pESCs and piPSCs, cells were fixed with 4% paraformaldehyde for 15 min. After washing, fixed cells were stained with a solution containing nitro blue tetrazolium chloride (NBT) and 5-bromo-4-chloro-3-indolyl phosphate toluidine salt (BCIP) stock solution (Roche, Madison, WI, www.roche.com) in a buffer solution for 30 min at room temperature. For ICC analysis of undifferentiated or differentiated cells, fixed cells were washed and permeabilized (for intracellular markers only) with 0.2% Triton X-100 (Sigma, USA, www.sigmaaldrich.com) for 5 min. Washed cells were co-incubated with blocking solution (10% goat serum in PBS) and a primary antibody overnight at 4°C. The primary antibodies used were Oct4 (SC-9081, Santa Cruz Biotechnology, www.scbt.com 1∶100), Nanog (SC-33759, Santa Cruz Biotechnology, 1∶100), Sox2 (AB5603, Millipore, Temecula, CA, www. millipore.com, 1∶200), SSEA-4 (MAB4304, Millipore, 1∶200), Tra 1–60 (MAB4360, Millipore, 1∶200), Tra 1–81 (MAB4381, Millipore, 1∶200), Neurofilament (MAB1615, Milllipore, 1∶200), Desmin (MAB3430, Millipore, 1∶200) and Cytokeratin 17 (MAB1625, Millipore, 1∶200). The cells were then washed, incubated with the appropriate secondary antibodies and stained with Hoechst 33342 or PI. Stained cells were examined using a confocal microscope and a ZEN 2009 Light Edition (Carl Zeiss, Germany, www.zeiss.com).

### Embryoid Body (EB) Formation and *in vitro* Differentiation

To evaluate differentiation potential, EpiSC-like pESCs and piPSCs were removed from MEFs, mechanically dissociated with glass pipettes and cultured in pESC medium without cytokines using the hanging drop method. After five days, EpiSC-like pESCs and piPSCs formed typical EBs, which were transferred to confocal dishes coated with 0.1% gelatin and allowed to further differentiate during 2–3 weeks of culture.

### Reverse Transcriptase-polymerase Chain Reaction (RT-PCR) Analysis and Real-time PCR

To analyze the gene expression patterns of undifferentiated or differentiated cells, total RNA from individual samples was extracted using TRIZOL® reagent (Invitrogen) according to the manufacturer’s instructions. cDNA was synthesized using a High Capacity RNA-to-cDNA Kit (Applied Biosystems, Forster City, CA, www.appliedbiosystems.com) according to the manufacturer’s instructions, producing a final volume of 20 µl. PCR amplifications were performed using a 2x PCR Master Mix Solution (i-MAX II, iNtRON Biotechnology) with a total reaction volume of 20 µl, containing 1 µl cDNA, 1 µl of each primer, and 7 µl distilled water. The conditions and primers used are listed in Table 1. Real-time PCR amplification was conducted using the ABI 7300 Real time PCR System (Applied Biosystems). A QuantiTect SYBER Green PCR kit (Finnzymes, Espoo, Finland, www.finnzymes.fi) was used to provide real-time quantification of the desired PCR product. The real-time PCR reaction mixture was comprised 1 µl cDNA, and 2 µl of each primer (Table 1) in a total volume of 20 µl. Four replications were conducted and the mRNA level of each sample was normalized to that of b-actin mRNA level. The relative levels of mRNA were analyzed by the delta-Ct method [37].

**Table 1:** Primers and conditions for RT-PCR.

Gene	Primer sequences	AnnealingTemperature (°C)	Productsize (bp)	AccessionNumber
*OCT4*	5′-AACGATCAAGCAGTGACTATTCG-3′	60	153	AF074419
	5′-GAGTACAGGGTGGTGAAGTGAGG-3′			
*NANOG*	5′-AATCTTCACCAATGCCTGAG-3′	60	141	DQ447201
	5′-GGCTGTCCTGAATAAGCAGA-3′			
*SOX2*	5′-CGGCGGCAGGATCGGC-3′	60	113	EU519824
	5′-GAGCTCCGCGAGGAAAA-3′			
*TDGF1*	5′-CAGGAGGAGCCTGCAATTCG-3′	60	101	TC207301
	5′-CCCCCATTCAGACAGCAGGT-3′			
*REX1*	5′-TTTCTGAGTACGTGCCAGGC-3′	60	201	TC206552
	5′-GAACGGAGAGATGCTTTCTCAGAG-3′			
*bFGF*	5′-GCGACCCTCACATCAAACT-3′	55	214	AJ577089.1
	5′-CAGTGCCACATACCAACT-3′			
*FGFR1*	5′-ACTGCTGGAGTTAATACCACCG-3′	55	125	AJ577088
	5′-GCAGAGTGATGGGAGAGTCC-3′			
*FGFR2*	5′-GGTGTTAACACCACGGACAA-3′	55	139	AJ439886.1
	5′-CTGGCAGAACTGTCAACCAT-3′			
*Activin-A*	5′-TGCGCATTGACATGTACGCC-3′	60	136	NM001005350
	5′-AGCTCCTCCAAGGACGGGTG-3′			
*NODAL*	5′-ATCAGGTCCCACCCGACTGC-3′	60	142	XM001928024
	5′-AGCTCCCCAGGGTGCTTCAG-3′			
*C-kit Ligand*	5′-GTTGGATAAGCGAAATGGTGG-3′	60	320	L07786
	5′-GTGACACTGACTCTGGAATCTTTT-3′			
*C-kit Receptor*	5′-CCTGGGATTTTCTCTTCGTC-3′	60	341	FJ938289
	5′-GACGAGGAAAAGCTTCTCAGG-3′			
*AFP*	5′-CGCGTTTCTGGTTGCTTACAC-3′	60	483	NM214317
	5′-ACTTCTTGCTCTTGGCCTTGG-3′			
*CRABP2*	5′-CTGACCATGACGGCAGATGA-3′	60	185	NM001164509
	5′-CCCCAGAAGTGACCGAAGTG-3′			
*DES*	5′-CCTCAACTTCCGAGAAACAAGC-3′	60	108	NM1001535
	5′-TCACTGACGACCTCCCCATC-3′			
*XIST*	5′-GAAGCATCAGCCAGCAACAC-3′	58	82	AJ429140
	5′-TCATAACCATCACTAGTACCCAAACC-3′			
*XIST BS*	5′-TAAGAAGTAGGATGGTTTAAGGAAGG-3′	58	210	UCSC Genome Bioinformatics^a^
	5′-CAACAAACAAAAACACCAACAATAC-3′			
*ß-ACTIN*	5′-GTGGACATCAGGAAGGACCTCTA-3′	60	137	U07786
	5′-ATGATCTTGATCTTCATGGTGCT-3′			

a The exact information was referred by materials and methods.

### Flow Cytometric Analysis

Colonies of EpiSC-like pES and piPS cells were mechanically detached from MEFs. After washing, detached colonies were dissociated into single cells using TrypLE™ Express (Gibco). Dissociated cells were fixed, permeabilized and incubated with primary antibodies overnight at 4°C. The primary antibodies used were Oct4 (Santa Cruz Biotechnology, 1∶10), Nanog (Santa Cruz Biotechnology, 1∶10), and Sox2 (Millipore, 1∶20). After washing, the cells were incubated with a suitable FITC-labeled secondary antibody. All samples were single-color stained using 10,000 cells for each marker, and the resulting data were analyzed using FACScalibur and Cell Quest software (BD Biosciences, CA, www.bd.com).

### DNA Isolation and Methylation Analysis

Isolation of genomic DNA from samples was carried out using G-spin Genomic DNA extraction kit (iNtRON) according to the manufacturer’s instructions. The bisulfite treatment of genomic DNA samples was performed with the EZ DNA Methylation-Gold™ kit (Zymo Reserch, USA, www.zymoresearch.com) according to the manufacturer’s instructions. The XIST promoter region of converted DNA samples was amplified using PCR with 2x PCR Master Mix Solution (iNtRON Biotechnology) and XIST BS primers listed in Table 1. The genomic data of XIST were derived from “UCSC Genome Bioinformatics” site (www.genome.ucsc.edu) and a converted primer set was designed by “Methprimer” program (www.urogene.org). PCR products were cloned into the pGEMT-Easy vector (Promega, WI, USA, www.promega.com) and transformed into E. coli cells (Novagen, USA) and at least 10 insert positive plasmid clones were sequenced by an ABI PRISM 3730 automated sequencer (Applied Biosystems). The methylation patterns were analyzed in sequences derived from clones.

### Microarray Analysis

Total RNAs from individual samples were extracted using RNeasy Mini Kit (QIAGEN, www.qiagen.com) based on the manufacturer’s instructions. The microarray data had been submitted to GEO database (GSE32506). To compare global gene expression of our pESCs with hESCs and mESCs, we downloaded the human and mouse cell information, hESCs (GSM628197-9, GSM525424-6), mESCs (GSM64922, GSM64924, GSM64926, GSM72622, GSM72624 and GSM72626) from NCBI GEO. We processed RMA of Affy (2.7) software package of the R statistical package to perform background subtracting and quantile normalization on each three species [38]. We made a gene expression orthologous set, by using microarray probe and orthologous information of pig (Sscrofa9), human (GRCh37.p3) and mouse (NCBIM37) from Ensembl Genes. We performed quantile normalization on 2606 orthologous probes using preprocess Core package [39]. The gplots package of the R statistical package was employed to generate heatmap plot [40].

### Karyotyping

Standard G-banding chromosome and cytogenetic analysis was carried out at Samkwang Medical Laboratories (Korea, www.smlab.co.kr).

### Statistical Analysis

Data are presented as the mean ± s.e.m. and were analyzed using Student’s t-test. All analyses were performed using R software. P<0.05 was considered significant.

## Results

### Generation of EpiSC-like pESCs and piPSCs

**Figure 1:**
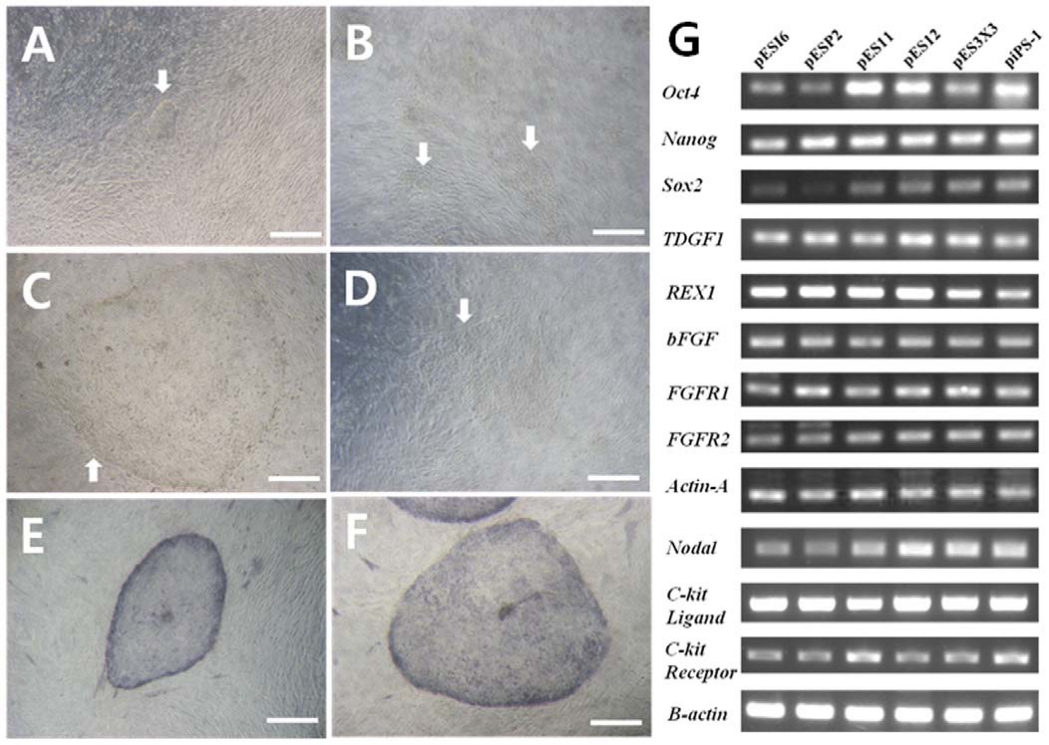
Derivation and gene expression analysis of porcine pluripotent cells. (**A**) Early morphology of EpiSC-like primary pESCs derived from porcine blastocysts. (**B**) Early morphology of EpiSC-like primary piPSCs derived from porcine embryonic fibroblasts. (**C**) Late morphology of EpiSC-like primary pESCs derived from porcine blastocysts. (**D**) Late morphology of EpiSC-like primary piPSCs derived from porcine embryonic fibroblasts. (**E**) AP activity of typical EpiSC-like pESCs. (**F**) AP activity of typical EpiSC-like piPSCs. (**G**) All of the pluripotent cells expressed the undifferentiated pluripotent stem cells markers Oct4, Sox2, Nanog, TDGF1 and Rex1 as well as the epiblast stem cell markers bFGF, FGFR1, FGFR2, Activin-A and Nodal. C-kit ligand and C-kit receptor, known growth factor for stem cell renewal, were also expressed in these cell lines. Scale bars = 200 µm.

We have been able to derive EpiSC-like pESC lines from various porcine blastocysts and a piPSC line from porcine embryonic fibroblasts. Information of our cell lines is summarized in Table 2. As it is difficult to identify and exclusively isolate ICM cells from intact *in vitro* produced blastocysts, we employed a method of whole blastocyst seeding directly onto MEFs to generate our pluripotent cell lines. Two to three days following the seeding of day 8 *in vitro* hatched blastocysts, giant cells originating from trophectoderm initially proliferated and then gradually died out or disappeared. After 5–7 days, we were able to observe primary colonies with an EpiSC-like morphology (Figure 1A). Primary colonies large enough to passage (Figure 1C) were mechanically dissociated into several clumps using pulled glass pipettes at 10–15 days post seeding. We could derive various cell lines from *in vitro* produced blastocysts using this method; however, we could not be entirely sure that these primary colonies originated from the ICM and not the trophectoderm.

**Table 2:** Information of derived cell lines and respective passages of cell lines used for each analysis in this study.

Cell line	Origin (porcine)	Initial culture method	Passages number§	Karyotype (passages)	No. of Passage		
					Flow cytometry	Immunocytochemistry	
						undifferentiation	differentiation
pESI6	*in vitro* fertilization (IVF) blastocysts	Whole Explant	>46	38 XX, normal (31)	15	26	33
pESP2	parthenogenetic blastocysts	Whole Explant	>42	38 XX, normal (22)	14	9	6
pES11	*in vivo* produced blastocysts	Whole Explant	>41	38 XX, normal (11)	16	15	14
pES12	*in vivo* produced blastocysts	Mechanical dissection	>45	38 XX, normal (25)	22	24	22
pES3X3	IVF aggregated (3X) blastocysts	Whole Explant	>45	38 XY, normal (12)	22	15	20
piPS-1	embryonic fibroblasts	Plasmid-transfection (four factors)	>55	38 XY, normal (7)	28	49	46

§ Derived cell lines could be maintained over the passage.

To determine whether these primary colonies represented ICM-derived cell lines, we used a mechanical dissection technique to eliminate trophectoderm cells prior to seeding. The cell line pES11 was derived using the whole blastocysts seeding method from day 7 *in vivo* produced blastocysts, whereas cell line pES12 was derived using the mechanical dissection method. Following dissection, the dissected ICM and trophectoderm were plated onto separate culture plates and cultured under the same culture conditions. We found that it was only possible to derive primary colonies from ICM tissues; trophectoderm seeding onto feeder cells routinely failed to generate any primary colonies, with cells initially proliferating before dying out or disappearing (data not shown). To avoid controversies regarding the origins of pluripotent cell lines, we compared the characteristics of pluripotent cell lines derived from whole blastocysts with those of cell line pES12 derived from ICM tissue alone. The results showed that our pluripotent cell lines derived through the whole seeding method are similar to both cell line pES12 and cell line piPS-1. This whole seeding method has been previously used to generate pluripotent cell lines from *in vitro* produced porcine embryos as it is often difficult to distinguish between ICM and trophectoderm tissues in these embryos [41]. Moreover, Heins et al. also reported the establishment of hESCs from hatched human blastocysts via whole seeding, showing that this is a viable pluripotent cell derivation technique [42]. Our results further demonstrate the usefulness of the whole seeding method in establishing pluripotent stem cell lines from *in vitro* produced porcine blastocysts. All of the cell lines derived from various embryos, including pES11 and pES12, showed similar primary colonies with typical morphology and AP activity, as shown in Figures 1A, C, and E.

We also attempted to derive piPSC line from PEFs via plasmid transfection using four reprogramming factors (*Oct4*, *Sox2*, *Klf4* and *c-MYC*). After transferring onto feeder cells, the PEFs with introduced reprogramming factors gradually demonstrated outgrowth, as shown in Figure 1B. We observed cells with an EpiSC-like morphology 2–3 weeks after seeding (Figure 1D). Cell line piPS-1 was derived via plasmid transfection and was routinely passaged every 5–7 days using pulled glass pipettes. The cell line piPS-1 displayed typical EpiSC-like morphology and AP activity (Figure 1F). In preliminary experiment, it was identified that transgenes (OKSM) were integrated into the genome of piPS-1.

Following the initial subculture of primary colonies, we used a mechanical dissociation method with pulled glass pipettes for routine subculture. When attempting to subculture our cells using enzymatic dissociation, we found that the proliferation efficiency and AP activity of these lines were significantly reduced (data not shown). These features are consistent with those of human or primate ES cells [43]. Therefore, a mechanical dissociation method is essential for the stable and long-term maintenance of EpiSC-like pESCs and piPSCs.

**Figure 2:**
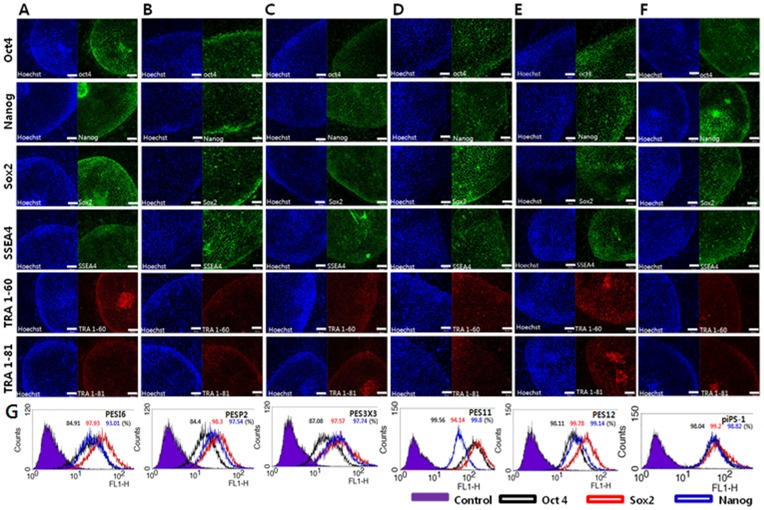
Expression of pluripotent markers in porcine pluripotent cells by immunocytochemistry analysis. Expression of pluripotent markers and surface markers Oct4, Sox2, Nanog, SSEA4, TRA 1–60 and TRA 1–81 was observed all six cell lines (**A**) pESI6, (**B**) pESP2, (**C**) pES3X3, (**D**) pES11, (**E**) pES12 and (**F**) piPS-1. Lanes to the left represent hoechst staining. (**G**) Expression of pluripotent markers Oct4, Sox2 and Nanog was detected in 84.91%, 97.93% and 93.01% of cells from the cell line pESI6, 84.4%, 98.3% and 97.54% in the cell line pESP2, 87.08%, 97.57% and 97.74% in the cell line pES3X3, 99.56%, 94.14% and 99.8% in the cell line pEpiS11, 98.11%, 99.78% and 99.14% in the cell line pES12 and 98.04%, 99.2% and 98.82% in the cell line piPS-1. Scale bars = 100 µm.

All cell lines maintained stemness characteristics (Figure 1G, 2) and a stable morphology for more than 45 passages, as shown in Table 2, and were cryopreserved in liquid nitrogen using EM grids. After thawing, all six lines proliferated without loss of stemness or normal morphology.

### Verification of Pluripotency Markers in EpiSC-like pESCs and piPSCs

We investigated the protein and mRNA expression levels of pluripotency markers in six cell lines that showed similar morphologies and AP activities. All of these cell lines expressed *Oct4*, *Sox2*, *Nanog, TDGF1* and *Rex1,* which are known early embryonic and undifferentiated pluripotency cell markers (Figure 1G). We also identified the expressions of *bFGF*, *FGFR1*, *FGFR2*, *Activin-A* and *Nodal*, which are commonly activated in epiblast or epiblast stem cells [12], [25], [44], [45] in all lines. All of the pluripotent cell lines, including line piPS-1, were cultured in the presence of hrbFGF and hrSCF as described in Materials and Methods. The addition of hrSCF to culture medium containing hrbFGF showed a greater positive effect on the stable maintenance of porcine pluripotent cells than did the addition of hrbFGF alone (data not shown), although further study is required to identify whether the addition of hrSCF is essential for the maintenance of stemness in porcine pluripotent cells. Interestingly, we found that mRNA expression of the *c-kit* ligand was high in all six cell lines (Figure 1G). This may be because of autocrine or paracrine effects among porcine pluripotent cells or to a synergistic effect of hrSCF itself, although factors secreted from the feeder cells should also be considered.

We investigated the expressions of the pluripotency and surface markers Oct4, Sox2, Nanog, SSEA4, TRA 1–60 and TRA 1–81 in all six cell lines, since expressions of these markers are a feature of hESCs [1]. As shown in Figure 2A–F, expressions of these markers were confirmed in all cell lines.

We also measured the relative expression levels of the pluripotency markers Oct4, Sox2 and Nanog among cell populations within the six cell lines using flow cytometric analysis. As a negative control, the cell lines were prepared without primary antibodies. All cell lines showed high expression levels of the pluripotent markers Oct4, Sox2 and Nanog. The results shown in Figure 2G were single-color stained, analyzed using FACScalibur with 10,000 cells per marker and overlapped using Cell Quest software (BD Biosciences, CA). As shown in Figure 2G, the pluripotency markers Oct4, Sox2 and Nanog were expressed in 84.91%, 97.93% and 93.01% of cells in the cell line pESI6; 84.4%, 98.3% and 97.54% of the cells in pESP2; 87.08%, 97.57% and 97.74% of pES3X3; 99.56%, 94.14% and 99.8% of pES11; 98.11%, 99.78% and 99.14% of pES12; and 98.04%, 99.2% and 98.82% of piPS-1.

### Epigenetic Characteristics of EpiSC-like pESCs and piPSCs, and Global Gene Expression Profile of EpiSC-like pESCs

X chromosome inactivation have been addressed as a hallmark to determine whether pluripotent cell is naïve or primed state. [10] Here, we could identify X chromosome inactivation status in our porcine pluripotent stem cell lines. X chromosome inactivation as a feature of primed state is related to the activation of *XIST* gene and the demethylation of *XIST* promoter.

**Figure 3:**
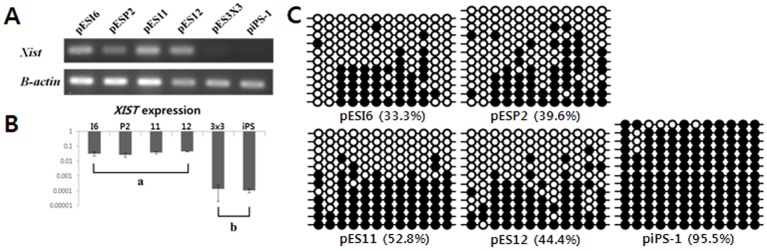
Epigenetic characteristics of EpiSC-like pESCs and piPSCs. (**A**) Investigation of the XIST gene expression from all cell lines. The result showed the expression of XIST gene in cell line pESI6, pESP2, pES11 and pES12 which are all female. Meanwhile, no expression was identified in male cell lines, pES3X3 and piPS-1. (**B**) Relative expression levels of XIST gene in individual cell lines clearly demonstrated the XIST gene in female cell lines was highly expressed comparable to male cell lines. Y-axis is expressed as a relative gene expression level in the cell lines. Data are represented as mean ± SEM (n = 5); Bars with different letters (a and b) are significantly different (P<0.05). (**C**) DNA methylation status of XIST promoter regions in female cell lines pESI6, pESP2, pES11 and pES12 and in male cell line piPS-1. Circles indicated the CpG sites of region analyzed. Open and closed circles mean unmethylation and methylation status.

First, we investigated the expression of *XIST* gene from all our cell lines using RT-PCR. As shown in Figure 3A, we could identify the expression of *XIST* gene in cell line PESI6, PESP2, PES11 and PES12 which are all female. Meanwhile, no expression was identified in male cell lines, PES3X3 and piPS-1. We next assessed relative expression levels of *XIST* gene in individual cell lines by real-time PCR. The results clearly demonstrated that the *XIST* gene in female cell lines was highly expressed when compared to male cell lines (Figure 3B). Finally, we confirmed DNA methylation status of XIST promoter regions in female cell lines pESI6, pESP2, pES11 and pES12. The result shown that XIST promoter was 33.3∼52.8% methylated in female cell lines, while it was 95.5% methylated in male cell line piPS-1 (Figure 3C).

**Figure 4:**
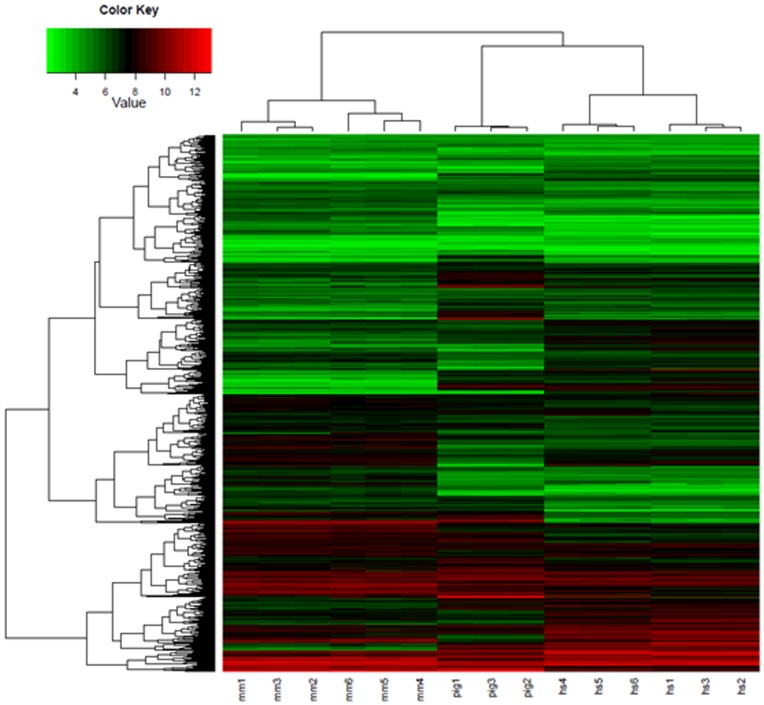
Global gene expression profile of EpiSC-like pESCs. Each row represents the expression of a single gene and columns indicate samples; mESCs, pESCs and hESCs. The 3 large groups in the heatmap were identified. Hierarchical clustering result showed the global gene expression pattern of pESCs is similar to hESCs. mm 1, 2 and 3; cell line R1, mm 4, 5 and 6; J1, pig 1, 2 and 3; cell line PES5, hs 1, 2 and 3 : cell line HUES6, hs 4, 5 and 6 : cell line H9.

To compare global gene expression of our pESCs with hESCs and mESCs, we conducted microarray analysis on pESCs derived from in vivo produced embryos using the GeneChip® Porcine Genome Array. In Figure 4, we could identify the 3 large groups in the heatmap. Hierarchical clustering result showed the global gene expression pattern of pESCs is more similar to that of hESCs than to mESCs.

### Differentiation Potential and Karyotyping of EpiSC-like pESCs and piPSCs

**Figure 5:**
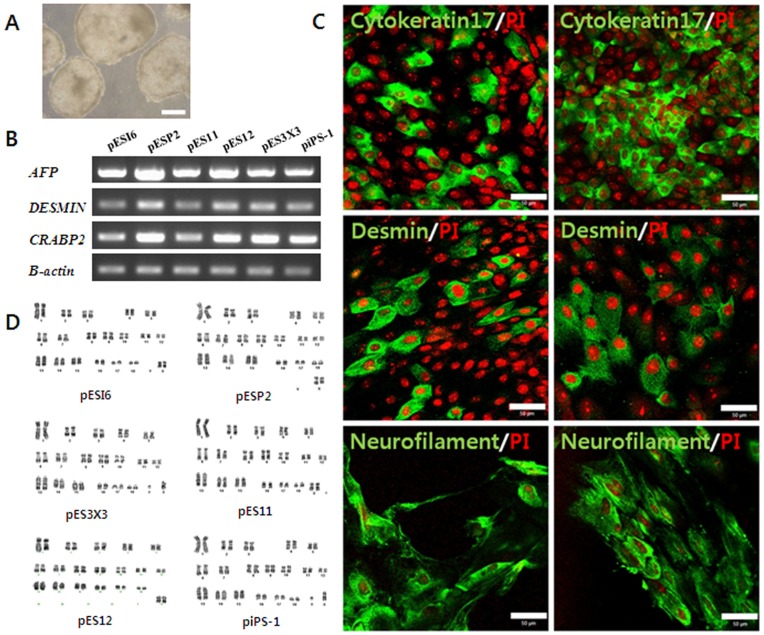
Differentiation potential and karyotyping of EpiSC-like pESCs and piPSCs. (**A**) Representative embryoid bodies derived from EpiSC-like pES cell lines and piPS cell lines through the culture for 5 days by hanging drop method. (**B**) When EBs cultured continuously onto culture plates, a variety of differentiated cells was observed. We could detect the expression of marker genes AFP (endoderm), DESMIN (mesoderm) and CRABP2 (ectoderm) involving differentiation. (**C**) Confirmation of the expression of differentiation marker Cytokeratin 17 (endoderm), Desmin (mesoderm) and Neurofilament (ectoderm) from differentiated cells by the immunocytochemistry analysis. Left lane is the cells differentiated from EpiSC-like pESCs and right lane is the cells differentiated from EpiSC-like piPSCs. (**D**) Both EpiSC-like pESCs and piPSCs have a normal karyotyping. Scale bars = 50 µm.

We investigated the differentiation potentials of the six cell lines to formation of EB and all three endodermal, mesodermal and ectodermal germ layers. As shown in Figure 5A, all cell lines showed the potential to undergo EB formation following five days of culture using the hanging drop method.

When EBs were cultured onto plates coated with 0.1% gelatin, a variety of differentiated cells were observed after 2–3 weeks (data not shown). We confirmed the expressions of three genes known to be involved in differentiation: *AFP* (endoderm), *DESMIN* (mesoderm), and *CRABP*2 (ectoderm). As shown in Figure 5B, all of the differentiation markers were expressed in all cell lines. We also investigated the expressions of differentiation markers from differentiated cells using immunocytochemical analysis. As shown in Figures 5C and 5D, both EpiSC-like pESCs and piPSCs not only expressed the differentiation markers Cytokeratin 17 (endoderm), Desmin (mesoderm), and Neurofilament (ectoderm), but also demonstrated normal karyotyping.

## Discussion

Several studies have been conducted with the aim of establishing ESC lines from porcine embryos of various origins [7], [15], [18]–[24]. However, most researchers to date have found it difficult to maintain an ES-like state in derived cell lines, with the cells showing a strong tendency to differentiate into an epithelial or EpiSC-like state [46], [47]. Accordingly, the purposes of present study are not only establishing pluripotent stem cells in pig, but also the comprehensive comparison of porcine pluripotent stem cells derived from various embryonic origin (*in vivo* embryos, *in vitro* produced embryos, parthenotes) and iPSCs in the aspect of pluripotent status. In addition, we expected to explain why authentic ESCs have not yet been categorized for many years in pigs. We have been able to derive cell lines of an EpiSC-like state from porcine embryos of various origins and could be stably maintained for long period, saying more than 1 year. All of our cell lines could be described as similar to epiblast stem cells with respect to their morphology and signaling pathways for the maintenance of pluripotency. Previous studies have shown Oct4, Rex1 and TDGF1 to be relatively good markers of porcine epiblast cells [48], while Sox2, Nanog, bFGF, FGFR1 and FGFR2 are exclusively expressed in porcine epiblast cells [44]. Consistent with previous reports, our results showed that all of our cell lines express the pluripotency genes Oct4, Sox2, Nanog, Rex1 and TDGF1 in addition to bFGF, FGFR1, FGFR2, Nodal and Activin-A, which are involved in signaling pathways activated in epiblast stem cells or hES cells. Moreover, our results showed X chromosome inactivation in our female cell lines is consistent with that of mEpiSC [49] (Figure 3). Therefore, we concluded that our cell lines derived from porcine embryos of various origins belong to the category of EpiSC-like ESCs rather than authentic ESCs.

Recently, several studies have reported the establishment of iPSC lines from porcine somatic cells using reprogramming factors [26], [27], [29]–[32]. All of these cell lines displayed characteristics attributed to primed pluripotent state cells, with flattened morphologies and FGF and Nodal/Activin signaling pathways similar to mEpiSCs and hESCs. We have also generated piPSCs from PEFs via plasmid transfection using four reprogramming factors (*Oct4*, *Sox2*, *Klf4* and *c-Myc*) and have derived cell lines of a primed state. In addition, our results suggest that cell line piPS-1 is very similar to all of our EpiSC-like pESC lines with respect to morphology, AP activity, the activation of signaling pathways and the expressions of pluripotency markers. Therefore, it can be concluded that piPSC is analogous to EpiSC-like pESCs, and both piPSC and EpiSC-like pESCs possess a primed pluripotent state rather than a naïve state. Previous reports demonstrated that patterns of gene expression of hESCs share those of mEpiSCs and clustered closely with those of mEpiSCs than to those of mESCs [50], [51]. Our cluster analysis of global gene expression showed that the global gene expression pattern of EpiSC-like pESCs is similar to hESCs, although further analysis is required to confirm this (Figure 4).

To investigate *in vivo* differentiation potential, all of our EpiSC-like ESC lines were injected subcutaneously into non-obese diabetic/severe combined immunodeficient (NOD/SCID) mice. Despite numerous attempts using increased cell numbers (1×10^7^ from 1×10^6^ EpiSC-like pESCs), we were unable to observe teratoma formation with any of our EpiSC-like pESC lines. We do not know why teratoma formation has so far remained impossible with our EpiSC-like pESC lines; however, to date, there are no reports of definite teratoma formation using pluripotent cell lines derived from porcine embryos. Anderson et al. have reported that it is difficult to generate embryonic carcinoma (EC) cells using early stage porcine embryos, unlike in mice and humans [52]. We presume that the failure of our EpiSC-like pESC lines to result in teratoma formation reflects the myriad of difficulties faced in EC derivation from porcine species.

Recent studies have reported that pluripotent stem cells exist in one of two forms and can be categorized according to their pluripotent state [10], [12]. The first is a “naïve” state, which represents full pluripotency or a ground state. The mESCs or mouse embryonic germ cells (mEGCs) could be categorized as such. The second “primed” state has been described as a state of limited pluripotency, within which mEpiSCs or hESCs can be placed. Nichols and Smith (2009) [10] have suggested that the establishment of the naïve state, which is full pluripotency, is species-dependent, and that permissive species are able to maintain a naïve state during stabilization into stem cell lines from naïve epiblast during preimplantation embryo development. In contrast to naïve state species, non-permissive species do not easily maintain a naïve state during the establishment of pluripotent cell lines and instead are stabilized into a primed state from naïve epiblasts.

The differences in the establishments of these pluripotent states result in many differences between the two states. For example, naïve state cell lines form small, round or dome-shaped colonies, with LIF and BMP4 signaling pathways playing an important role in the maintenance of pluripotency. Furthermore, both X chromosomes remain activated (X^a^X^a^) in female cells and the *Oct4* distal enhancer is still active in naïve state cell lines, as can be seen in early embryonic cells. In contrast, primed pluripotent cell lines display flattened monolayer colony morphologies and show activations of FGF and Nodal/Activin signaling pathways for maintenance of pluripotency, X chromosome-inactivation (X^a^X^i^) and activation of the proximal element of the *Oct4* enhancer, which represent distinct similarities with primed epiblast cells of post-implantation embryo stage development [10], [12]. Interestingly, when pluripotent stem cell lines are established from non-obese diabetic (NOD) mice or rats, which are non-permissive species [53], primed pluripotent cell lines can be derived from blastocyst stage embryos and induced from somatic cells [11].

Recent studies have reported that primed pluripotent stem cell lines could be reverted to a naïve pluripotent state using various exogenous factors including GSK3β and MEK inhibitors, LIF, hypoxic conditions and up-regulation of klf4 [11], [51], [54]–[56]. Recently, Roberts and colleagues reported LIF-dependent pluripotent stem cells derived from porcine embryos by up-regulation of Oct4 and Klf4 [57]. We are also trying to create a LIF-dependent naïve pluripotent porcine stem cell line using various exogenous factors and have so far been able to successfully induce PEFs into a naïve pluripotent cell line showing a mESC-like morphology. In the future, we intend to investigate the potential of germ cell specification through the use of chimeras and the comparison of epigenetic states, such as X chromosome and Oct4 enhancer activation, between naïve pluripotent cell lines and primed pluripotent cell lines in pig.

In conclusion, we have been able to derive EpiSC-like pESC lines from porcine embryos of various origins including *in vitro* fertilized (IVF), *in vivo* derived, IVF aggregated (3X) and parthenogenetic embryos, in addition to a piPSC line derived from PEFs, via plasmid transfection with reprogramming factors (*Oct4*, *Sox2*, *Klf4* and *c-Myc*). All of our cell lines showed AP activity and expressions of the genes *Oct4*, *Sox2*, *Nanog*, *Rex1*, *TDGF1*, *bFGF*, *FGFR1*, *FGFR2*, *Nodal* and *Activin-A* involved in pluripotency and signaling pathways, X chromosome inactivation in female cell lines, in vitro differentiation potential and a normal karyotype, thus displaying similarities to mEpiSCs or hESCs, which was supported by the comparison of the global gene expression pattern. Therefore, our data suggest that, as a non-permissive species, the porcine embryos and somatic cells undergoes reprogramming into a primed state during the establishment of pluripotent stem cell lines based on genomic background. Our results will help not only to understand the reprogramming into a primed state during the establishment of pluripotent stem cell lines in pigs, but also explain current status of stem cell research in domestic animals.
